# Evaluating Genome-Wide Association Study-Identified Breast Cancer Risk Variants in African-American Women

**DOI:** 10.1371/journal.pone.0058350

**Published:** 2013-04-08

**Authors:** Jirong Long, Ben Zhang, Lisa B. Signorello, Qiuyin Cai, Sandra Deming-Halverson, Martha J. Shrubsole, Maureen Sanderson, Joe Dennis, Kyriaki Michailiou, Douglas F. Easton, Xiao-Ou Shu, William J. Blot, Wei Zheng

**Affiliations:** 1 Division of Epidemiology, Department of Medicine, Vanderbilt Epidemiology Center, Vanderbilt-Ingram Cancer Center, Vanderbilt University School of Medicine, Nashville, Tennessee, United States of America; 2 International Epidemiology Institute, Rockville, Maryland, United States of America; 3 Meharry Medical College, Nashville, Tennessee, United States of America; 4 Centre for Cancer Genetic Epidemiology, Department of Public Health and Primary Care, University of Cambridge, Cambridge, UK; 5 Centre for Cancer Genetic Epidemiology, Department of Oncology, University of Cambridge, Cambridge, UK; IFOM, Fondazione Istituto FIRC di Oncologia Molecolare, Italy

## Abstract

Genome-wide association studies (GWAS), conducted mostly in European or Asian descendants, have identified approximately 67 genetic susceptibility loci for breast cancer. Given the large differences in genetic architecture between the African-ancestry genome and genomes of Asians and Europeans, it is important to investigate these loci in African-ancestry populations. We evaluated index SNPs in all 67 breast cancer susceptibility loci identified to date in our study including up to 3,300 African-American women (1,231 cases and 2,069 controls), recruited in the Southern Community Cohort Study (SCCS) and the Nashville Breast Health Study (NBHS). Seven SNPs were statistically significant (*P*≤0.05) with the risk of overall breast cancer in the same direction as previously reported: rs10069690 (5p15/*TERT*), rs999737 (14q24/*RAD51L1*), rs13387042 (2q35/*TNP1*), rs1219648 (10q26/*FGFR2*), rs8170 (19p13/*BABAM1*), rs17817449 (16q12/*FTO*), and rs13329835 (16q23/*DYL2*). A marginally significant association (*P*<0.10) was found for three additional SNPs: rs1045485 (2q33/*CASP8*), rs4849887 (2q14/*INHBB*), and rs4808801 (19p13/*ELL*). Three additional SNPs, including rs1011970 (9p21/*CDKN2A/2B*), rs941764 (14q32/*CCDC88C*), and rs17529111 (6q14/*FAM46A*), showed a significant association in analyses conducted by breast cancer subtype. The risk of breast cancer was elevated with an increasing number of risk variants, as measured by quintile of the genetic risk score, from 1.00 (reference), to 1.75 (1.30–2.37), 1.56 (1.15–2.11), 2.02 (1.50–2.74) and 2.63 (1.96–3.52), respectively, (*P* = 7.8×10^–10^). Results from this study highlight the need for large genetic studies in AAs to identify risk variants impacting this population.

## Introduction

Breast cancer is one of the most common malignancies diagnosed among women worldwide, including women of African descendent. African American (AA) women experience a disproportionate burden of breast cancer. Age-adjusted mortality rate of this cancer is more than 40% higher in AAs than in European-ancestry populations. AA women tend to be diagnosed with breast cancer at a younger age and with more aggressive types of the disease, such as ER- (estrogen receptor negative) and ER−/PR−/HER2- (estrogen receptor negative, progesterone receptor negative, HER2 expression negative) breast cancer. Within AAs, women having a higher African ancestry level, estimated by ancestry informative markers (AIMs), have been shown to have an increased likelihood of ER−/PR- versus ER+/PR+ breast cancers [1]. However, such association was not observed in the Women’s Contraceptive and Reproductive Experiences (CARE) study [2].

To date, four high-penetrance genes (*BRCA1, BRCA2, TP53*, and *PTEN*) and four moderate-penetrance genes (*CHEK2, ATM, BRIP1*, and *PALB2*) have been discovered for breast cancer [3]. Candidate gene studies have largely failed to identify low-penetrance loci which can be robustly replicated in other studies [3]. Genome-wide association studies (GWAS) have emerged as the most widely used approach to identify genetic variants for complex diseases [4]. Since 2007, 67 common genetic susceptibility loci have been discovered, including 25 from several earlier GWAS [5]–[21] and 42 from a recent international Collaborative Oncological Gene-Environment Study (COGS) [22]. However, except the 5p15/TERT locus which was discovered among AA women [20], all other risk variants initially were identified in studies conducted in European or Asian descendants. Given the considerable differences in genetic architecture, including allele frequencies, linkage disequilibrium (LD) structure, and genetic diversity between the African-ancestry genome and genomes of Asian- and European-ancestry populations [23], it is important to investigate whether GWAS-identified variants are associated with breast cancer risk in African-ancestry populations. This investigation not only assesses the generalizability of initial GWAS findings, but also provides valuable data to guide fine-mapping efforts in the search for causal variants.

In this current study, we evaluated risk variants in all 67 breast cancer loci identified to date in an AA population of 1,231 cases and 2,069 controls.

## Materials and Methods

This study uses resources from the Southern Community Cohort Study (SCCS) and the Nashville Breast Health Study (NBHS). The NBHS is a population-based case-control study [10]. Incident breast cancer cases were identified through the Tennessee State Cancer Registry and a network of major hospitals that provide medical care for patients with breast cancer. Controls were identified mostly via random-digit dialing of households in the same geographic area as cases and frequency-matched to cases on age (5-year group). All participants were phone-interviewed to obtain information related to personal and family medical history, and other lifestyle factors. A total of 437 cases and 252 controls from the NBHS who provided exfoliated buccal cell samples were included in the project. The SCCS is a prospective cohort study initiated in 2002 investigating racial disparities in the risk of cancer and other chronic diseases [24]. SCCS includes approximately 86,000 participants with two-thirds being AAs. In the SCCS, participants completed a comprehensive, in-person, baseline interview or completed a study questionnaire covering various aspects of health conditions, behavioral factors, personal and family medical history, and other lifestyle factors. In the SCCS, 679 breast cancer cases (217 incident and 462 prevalent cases) were included in the project. They were selected from those who were diagnosed with breast cancer and provided a blood or buccal cell sample. In the SCCS, controls (*n* = 680) were selected randomly from those who were cancer-free and frequency-matched to cases in a 1∶1 ratio on age at enrollment (±1 year), recruitment method, and sample type (blood/buccal cell).

### Ethics Statement

Written, informed consent was obtained from all participants prior to interview, and the study protocols have been approved by Institutional Review Boards at Vanderbilt University (for NBHS and SCCS) and Meharry Medical College (for SCCS).

Genotyping for samples described above was conducted using the protocol for the COGS Project as described elsewhere [22]. In brief, a custom Illumina Infinium BeadChip which contains 211,155 SNPs was genotyped. Individuals were excluded for any of the following reasons: 1) genotypically not female, 2) call rate <95%, 3) low or high heterozygosity (*P*<10^–6^), 4) genotyping not concordant with previous data, 5) duplicates or “cryptic” duplicates, 6) first-degree relative, 7) ethnic outliers based on a subset of 37,000 uncorrelated markers which passed QC. SNPs were excluded for any of the following reasons: 1) call rate <95%, 2) deviated from HWE in controls at *P*<10^–7^, 3) genotyping discrepancies in more than 2% of duplicate samples. Data cleaning was conducted within the whole COGS Project. After QC, a total of 199,961 SNPs for 1,116 cases and 932 controls were included in the dataset. We then performed principal component analyses (PCA) using a set of 4,613 uncorrelated SNPs (neighboring distance >500 kb, MAF >0.2, *r* ^2^<0.1, and call rate >99%). Five additional participants (three cases and two controls) were excluded due to >6 σ away from the means of PCA1 and PCA2. In total, 1,113 cases and 930 controls from the SCCS and NBHS were successfully genotyped by the COGS SNP array.

In our previous project, nine GWAS-identified SNPs were genotyped using Taqman/Sequenom in 810 cases and 1,784 controls from the NBHS and SCCS [25]. Among them, 118 cases and 1,139 controls were not included in the study using COGS SNP array. Data for these nine SNPs, including rs13387042, rs10941679, rs889312, rs2046210, rs13281615, rs1219648, rs2981582, rs3817198, and rs3803662, were combined from the data genotyped through Taqman/Sequenom [25] and data newly obtained using COGS SNP array. In total, 1,231 cases and 2,069 controls from the SCCS (743 cases and 1,797 controls) and NBHS (488 cases and 272 controls) with genotype data available were included in the final analyses.

### Statistical Analysis

Individual African ancestry level was estimated from 612 AIMs included in the COGS SNP array using the program frappe (http://med.stanford.edu/tanglab/software/frappe.html), which implements an Estimation-Maximization algorithm for simultaneously inferring each individual’s ancestry proportion and allele frequencies in the ancestral populations [26]. Associations between individual SNP and breast cancer risk were assessed using odds ratios (ORs) and 95% confidence intervals (CIs) derived from logistic regression models and adjusted for age and study site. In the present study, data are available for ER, PR and Her2 among 564, 555, and 250 breast cancer women, respectively. Subgroup analyses were conducted within breast cancer subtypes including ER+, ER−, and ER−/PR−/HER2−. Principal component analyses (PCA) were conducted based on 4,613 uncorrelated SNPs using EIGENSTRAT [27]. The first ten principal components were included in the logistic regression model to test association for the SNPs in the present study.

To evaluate the combined effect of SNPs on breast cancer risk, we created a weighted genetic risk score (GRS) for each study participant by multiplying the number of risk alleles (0/1/2) of each SNP by the weight (log scale of the per-allele OR derived from the current study) for that SNP, and then summing them together. Since data for a complete set of SNPs were only available for the 1,110 cases and 929 controls genotyped using COGS SNP array, GRS analysis was conducted among these subjects.

We constructed a GRS using SNPs that showed a statistically significant association with breast cancer risk in this study. In the present study, ten SNPs were associated with breast cancer (*P*<0.1) with direction of association consistent with previous reports. SNP rs1219648 was not included in the COGS SNP array and only 66% women of the COGS subjects had data available for this SNP, genotyped in our previous project through Sequenom (23). SNP rs13387042 was genotyped in both by the COGS SNP array and Sequenom (23). This SNP, however, did not reach *P*<0.1 in the samples analyzed using COGS SNP array and thus was not included in the GRS analyses. Thus, these two SNPs, rs1219648 and rs13387042, were excluded in GRS analyses. The remaining eight SNPs, rs10069690, rs999737, rs8170, rs17817449, rs13329835, rs1045485, rs4849887, and rs4808801 were included in the GRS analyses. All statistical analyses were conducted in SAS, version 9.3, with the use of two-tailed tests.

## Results

The distributions of demographic characteristics and known breast cancer risk factors for cases and controls are shown in Table 1. Cases were more likely to have a family history of breast cancer, an earlier age at menarche, fewer live births, older age at first live birth and high body mass index.

**Table 1:** Characteristics of study participants.

Characteristic	Cases (N = 1,231)	Controls (N = 2,069)	P value
Sample sources			
NBHS^a^	488	272	
SCCS^b^	743	1,797	
Age (year, mean±sd)	54.83±9.74	55.14±9.64	0.37
Age at menarche (year, mean±sd)	12.60±1.87	12.83±2.04	0.002
Postmenopausal (%)	78.69	73.49	<0.001
Number of live births (median, range)	3 (1, 16)	3 (1, 18)	0.027
Age at first live birth (year, mean±sd)^c^	20.95±5.09	20.42±4.77	0.004
Breast cancer family history (%)	20.69	10.36	<0.001
BMI (kg/m^2^, mean±sd)	31.95±7.34	31.49±8.07	0.108
BMI in postmenopausal women	32.16±7.21	31.45±7.97	0.03

a The Nashville Breast Health Study.

b The Southern Community Cohort Study.

c Among parous women.

### African Ancestry Level and Breast Cancer

We did not find any statistically significant association between African ancestry level and breast cancer risk. On average, cases had 83.22% African ancestry, and controls had 83.86%. No significant association was observed between African ancestry level with breast cancer subtype, either. The African ancestry proportion was 82.50%, 82.74%, and 82.25% for ER+/PR+, ER−/PR−, ER−/PR−/HER2− cases, respectively (Table S1). However, difference was observed between the two study cohorts with a higher African ancestry level in the SCCS than in the NBHS. On average, the African ancestry level is 85.23% and 80.14% in controls, and 84.50% and 81.22% in cases from the SCCS and NBHS, respectively.

### Evaluation of SNPs in 25 Previously Reported Loci

Two SNPs have been discovered in the 10q21/*ZNF365* locus: rs10995190 in Europeans [21] and rs10822013 in East Asians [17]. These two SNPs are not in LD based on data from HapMap Africans (r^2^ = 0.001), and both of them were included in the present study. Similarly, in the locus 16q12/*TOX3,* SNPs rs4784227 and rs3803662 were discovered in Asians [14] and Europeans [7], respectively. SNP rs4784227 was not included in the COGS SNP array. SNP rs17271951, which was in strong LD with rs4784227 based on HapMap Africans (r^2^ = 1.0), was used as a substitute. SNPs rs17271951 and rs3803662 are not in LD in HapMap Africans (r^2^ = 0.03), and both of them were included in the final analyses. In the 10q26/*FGFR2* locus, both rs1219648 and rs2981582 were discovered in Europeans but were in moderate LD in HapMap Africans (r^2^ = 0.25). For the 6q25/*TAB2* locus, rs9485370 was used to replace the originally reported SNP rs9485372 [28] which was not included in the COGS SNP array (r^2^ = 1 in HapMap Africans). For the other 21 loci, one index SNP (reported in previous GWAS) per locus was selected. Therefore, in total, 28 SNPs in previously reported 25 loci were investigated in the present study. Five of them were deviated from Hardy-Weinberg test with P<0.05, including rs1045485, rs10995190, rs13281615, rs889312, and rs999737.

Of the 28 SNPs, 19 SNPs had an OR of breast cancer risk in the same direction as initial reports. This is higher than expected under the null hypothesis (*P* = 0.04, binomial sign test). Five SNPs were nominally statistically significant (*P*≤0.05) in the same direction as previously reported (Table 2 and Table S2). SNP rs10069690 in the 5p15/*TERT* locus, previously discovered in African-ancestry population, showed a moderate-strong association in the present study with OR (95% CI) 1.19 (1.05–1.36) and *P*-value 0.007. Notably, the MAF is very low (0.047 in controls) for SNP rs999737 at 14q24/*RAD51L1*, however, a strong association was observed for this SNP, with OR (95% CI) 1.59 (1.15–2.19) and *P*-value 0.005. Allelic OR (95% CI) for the other 3 SNPs are 1.17 (1.04–1.33) for rs13387042 (*P* = 0.011), 1.17 (1.04–1.33) for rs1219648 (*P* = 0.011), and 1.25 (1.07–1.47) for rs8170 (*P* = 0.006). The *CASP8* SNP (rs1045485) identified previously through a candidate gene approach has a low frequency (MAF = 0.065) in AAs. A marginally significant association was found for this SNP with OR (95% CI) of 1.25 (0.96–1.62) and *P*-value 0.096. Significant association was observed for SNP rs4973768 at 3p24/*SLC4A7* in this study, however, in the opposite direction as previously reported. No association was observed with the other 21 SNPs. Among them, the minor allele in rs10771399 at 12p11/*PTHLH* is very rare in AAs with MAF = 0.034 in controls.

**Table 2:** Associations of breast cancer risk with 10 SNPs located in reported breast-cancer susceptibility loci in African Americans with P<0.1.

SNP	Chr./gene^a^	Allele^b^	RAF (cases/controls)^c^	N (cases/controls)	OR (95% CI)^d^		*P*_trend_	Power (%)^e^
					Heterozygous	Homozygous		
rs1045485	2q33/CASP8	G/C	0.946/0.935	1,113/930	1.99 (0.57–6.93)	2.38 (0.70–8.04)	0.096	54
rs13387042	2q35/TNP1	A/G	0.762/0.739	1,230/2,059	1.14 (0.82–1.59)	1.35 (0.98–1.87)	0.011	86
rs10069690	5p15/TERT	T/C	0.629/0.592	1,112/930	1.39 (1.07–1.81)	1.50 (1.15–1.97)	0.007	86
rs1219648	10q26/FGFR2	G/A	0.456/0.416	826/1,769	1.12 (0.92–1.37)	1.39 (1.09–1.79)	0.011	83
rs999737	14q24/RAD51L1	C/T	0.966/0.953	1,113/930	2.08 (0.39–11.20)	3.24 (0.62–16.98)	0.005	97
rs8170	19p13/BABAM1	A/G	0.212/0.177	1,112/930	1.19 (0.98–1.44)	1.90 (1.15–3.12)	0.006	88
rs4849887	2q14/INHBB	C/T	0.725/0.697	1,113/930	1.15 (0.83–1.60)	1.30 (0.94–1.80)	0.075	52
rs17817449	16q12/FTO	T/G	0.636/0.597	1,113/930	1.02 (0.79–1.34)	1.37 (1.04–1.80)	0.004	92
rs13329835	16q23/DYL2	G/A	0.677/0.655	1,113/930	0.88 (0.65–1.20)	1.16 (0.86–1.58)	0.037	71
rs4808801	19p13/ELL	A/G	0.305/0.279	1,112/929	1.09 (0.90–1.31)	1.34 (0.96–1.85)	0.091	50

a The closest gene.

b Risk/reference alleles based on NCBI Human Genome Build 36 forward strand.

c Risk allele frequency of cases and controls.

d Adjusted with age, study (NBHS and SCCS), and the first ten principal components.

e Power to identify an association at an alpha level of 0.05 under additive model.

### Evaluation of SNPs in 42 Newly Identified Loci

Of the 42 SNPs, one for each of the 42 loci recently discovered in European-ancestry populations [22] were included in the present study. Among them, SNPs rs11780156, rs13329835, rs17356907, rs616488, and rs6762644 were deviated from HWE test with P<0.05. Among the 42 investigated SNPs, 27 had an OR of breast cancer risk in the same direction in Europeans (Table 2 and Table S2). However, only two of them showed a nominally statistically significant association (*P*≤0.05). The OR (95% CI) was 1.21 (1.06–1.37) for rs17817449 at 16q12/*FTO* (*P* = 0.004) and 1.16 (1.01–1.32) for rs13329835 at 16q23/*DYL2* (*P* = 0.037). A marginally significant association (*P*<0.10) in the same direction as in Europeans was found for two additional SNPs, with ORs (95% CI) of 1.13 (0.99–1.30) for rs4849887 at 2q14/*INHBBP* (*P* = 0.075), and 1.13 (0.98–1.29) for rs4808801 at 19p13/*ELL* (*P* = 0.091). The 22q13/*MKL1* SNP rs6001930 showed an association (*P* = 0.035), however the direction was inconsistent with that observed in Europeans. No associations were identified for the other 37 SNPs, among which four SNPs have a MAF <0.05, including rs11552449 at 1p13/*AP4B1*, rs1353747 at 5q11/*PDE4D*, rs11780156 at 8q24/*MYC,* and rs11571833 at 13q13/*BRCA2*.

### Evaluation of Associations by Breast Cancer Subtypes

Association results between SNPs with risk of breast cancer by subtypes, including ER+, ER-, and ER−/PR−/HER2−, are presented in Table 3. Only SNPs that showed significant (*P*≤0.05) or marginally significant (*P*≤0.1) association with any of these three subtypes of breast cancer are presented. Three SNPs that were not associated with overall breast cancer showed nominally statistical significance (*P*≤0.05) in analysis by subtypes in the same direction as previously reported. SNPs rs1011970 at 9p21/*CDKN2A/2B* and rs941764 at 14q32/*CCDC88C* were associated with ER+ breast cancer with ORs (95% CI) 1.27 (1.05–1.54) (*P* = 0.014) and 1.26 (1.02–1.56) (*P* = 0.032), respectively. SNP rs17529111 at 6q14/*FAM46A* was associated with ER−/PR−/HER2− tumor with OR (95% CI) of 1.97 (1.02–3.82) (*P* = 0.043). Differences in the strength of the association were also observed for three other SNPs across breast cancer subtypes. SNP rs17817449 at 16q12/*FTO* showed an association with ER+ tumor with OR (95% CI) of 1.32 (1.09–1.60) but not with ER− or ER−/PR−/HER2− tumors. Both rs10069690 at 5p15/*TERT* and rs999737 at 14q24/*RAD51L1* showed the strongest association for ER−/PR−/HER2− breast cancer though associations were also observed for ER+ and ER− cancers.

**Table 3:** Association of breast cancer risk with selected GWAS-identified SNPs located in reported breast-cancer susceptibility loci in African Americans, by breast cancer subtypes.

SNP^a^	Chr./Gene^b^	ER+ (N = 369)		ER- (N = 195)		ER−/PR−/HER2- (N = 68)		*P*_heterogeneity_ d
		OR (95% CI)^c^	*P*_trend_ c	OR (95% CI)^c^	*P*_trend_ c	OR (95% CI)^c^	*P*_trend_ c	
rs13387042	2q35/*TNP1*	1.20 (0.99–1.47)	0.064	1.10 (0.86–1.42)	0.441	1.00 (0.67–1.51)	0.985	0.591
rs10069690	5p15/*TERT*	1.18 (0.98–1.42)	0.075	**1.36 (1.07–1.72)**	**0.011**	**1.58 (1.07–2.32)**	**0.021**	0.359
rs9485370	6q25/*TAB2*	1.21 (0.97–1.52)	0.093	0.85 (0.65–1.10)	0.209	**0.70 (0.46–1.05)**	**0.082**	0.041
rs1011970	9p21/*CDKN2A/2B*	**1.27 (1.05–1.54)**	**0.014** e	0.96 (0.75–1.22)	0.733	1.08 (0.73–1.58)	0.700	0.074
rs999737	14q24/*RAD51L1*	1.39 (0.88–2.18)	0.156	**2.20 (1.11–4.36)**	**0.024**	4.72 (1.10–20.28)	0.037	0.270
rs17271951	16q12/*TOX3*	1.14 (0.79–1.66)	0.485	1.00 (0.62–1.62)	0.994	1.77 (0.95–3.31)	0.075	0.672
rs8170	19p13/*BABAM1*	**1.30 (1.04–1.64)**	**0.023**	**1.36 (1.03–1.81)**	**0.033**	1.51 (0.95–2.40)	0.079	0.815
rs4849887	2q14/*INHBB*	**1.23 (1.00–1.50)**	**0.045**	1.25 (0.96–1.61)	0.093	1.46 (0.95–2.24)	0.087	0.934
rs6762644	3p26/*ITPR1*	1.18 (0.99–1.41)	0.067	1.09 (0.87–1.36)	0.440	0.87 (0.60–1.25)	0.436	0.593
rs9790517	4q24/*TET2*	0.95 (0.64–1.40)	0.790	1.52 (0.98–2.35)	0.064	1.33 (0.65–2.70)	0.433	0.118
rs17529111	6q14/*FAM46A*	0.97 (0.67–1.42)	0.893	1.32 (0.84–2.07)	0.229	**1.97 (1.02–3.82)**	**0.043**	0.312
rs9693444	8p21/*RPL17P33*	0.96 (0.80–1.16)	0.675	1.21 (0.96–1.52)	0.099	1.32 (0.92–1.90)	0.131	0.122
rs11199914	10q26/*FGFR2*	1.00 (0.83–1.19)	0.965	1.09 (0.87–1.37)	0.444	1.38 (0.96–1.98)	0.078	0.532
rs941764	14q32/*CCDC88C*	**1.26 (1.02–1.56)**	**0.032**	0.92 (0.72–1.19)	0.536	0.89 (0.60–1.33)	0.570	0.063
rs17817449	16q12/*FTO*	**1.32 (1.09–1.60)**	**0.004**	1.15 (0.91–1.46)	0.235	0.98 (0.67–1.44)	0.931	0.378
rs1436904	18q11/*CHST9*	1.02 (0.83–1.27)	0.822	1.09 (0.83–1.44)	0.528	1.51 (0.92–2.47)	0.100	0.718
rs4808801	19p13/*ELL*	0.95 (0.78–1.17)	0.644	1.24 (0.97–1.59)	0.083	1.41 (0.95–2.11)	0.091	0.103

a Only SNPs that showed significant (P≤0.05) or marginally significant (P≤0.1) association with any of these three subtypes of breast cancer are presented.

b The closest gene.

c Adjusted with age, study (NBHS and SCCS), and the first ten principal components.

d *P* for heterogeneity across ER positive and negative cases was calculated using a Cochran’s *Q* test.

e results with P<0.05 were bolded.

### Genetic Risk Score (GRS) Analyses for Overall Breast Cancer

Significant associations were observed between GRS and risk of breast cancer (Table 4). ORs (95% CIs) for overall breast cancer risk across increasing quintiles of GRS were 1.00 (reference), 1.75 (1.30–2.37), 1.56 (1.15–2.11), 2.02 (1.50–2.74) and 2.63 (1.96–3.52) (*P* = 7.8×10^–10^). Such associations also were observed for all three subtypes of breast cancer.

**Table 4:** Association of genetic risk score (GRS) with breast cancer risk in African Americans.

GRS quintile^a^	Controls	All cases		ER+ cases		ER- cases		ER−/PR−/HER2- cases	
	N = 929	N = 1,110	OR (95% CI)^b^	N = 337	OR (95% CI)^b^	N = 188	OR (95% CI)^b^	N = 67	OR (95% CI)^b^
Q1	185	133	1.00 (reference)	38	1.00 (reference)	26	1.00 (reference)	10	1.00 (reference)
Q2	185	221	1.75 (1.30–2.37)	66	1.91 (1.20–3.02)	27	1.16 (0.64–2.09)	8	0.95 (0.36–2.54)
Q3	194	202	1.56 (1.15–2.11)	68	1.90 (1.20–2.99)	32	1.30 (0.74–2.30)	11	1.23 (0.50–3.06)
Q4	176	236	2.02 (1.50–2.74)	73	2.39 (1.51–3.77)	41	2.06 (1.19–2.57)	13	2.06 (0.84–5.05)
Q5	189	318	2.63 (1.96–3.52)	92	2.85 (1.83–4.45)	62	2.82 (1.68–4.75)	25	3.35 (1.48–7.54)
*P*_for trend_			7.8×10^–10^		5.2×10^–6^		4.3×10^–6^		4.5×10^–4^

a GRS was constructed based on 8 SNPs, including rs1045485, rs10069690, rs999737, rs8170, rs4849887, rs17817449, rs13329835 and rs4808801.

b Adjusted for age, study, and the first ten principal components.

## Discussion

In the present study, we investigated associations of 70 index SNPs in 67 breast cancer susceptibility loci identified to date in up to 1,231 cases and 2,069 controls of AA women. We found that seven SNPs were significantly associated (*P*<0.05) and three SNPs were marginally significantly associated (*P*<0.10) with overall breast cancer risk in the same association direction as previously reported. Three additional SNPs showed a significant association (P<0.05) when stratified by breast cancer subtype. GRS analyses showed significant associations with the risk of overall or subtype of breast cancer.

In the present study population, on average, approximate 83% of genetic ancestry is African origin, which is similar to the estimate in other studies [1], [2], [29], [30]. Women in the SCCS have a higher African ancestry level than those in the NBHS. In the NBHS, most women were recruited in Tennessee, while SCCS women were recruited in 12 southern states including Alabama, Arkansas, Florida, Georgia, Kentucky, Louisiana, Tennessee, Mississippi, South Carolina, North Carolina, Virginia, and West Virginia. We did not find a significant difference in African ancestry level between breast cancer cases and controls or across breast cancer subtypes. These results are consistent with the recent finding in the CARE Study [2]. However, in another study, significant association was observed between genetic ancestry with ER+, PR+, or localized breast tumors [1]. In the present study, data for ER, PR and HER2 were available for only a portion of the subjects. Therefore, statistical power in regard to subtype of breast cancer analyses may be limited.

Among the ten SNPs that showed significant or marginally significant association with overall breast cancer risk in the present study, six have been investigated in previous studies of African-ancestry populations [20], [25], [31]–[36]. SNP rs10069690 (5p15/*TERT*) was originally discovered in a GWAS of AAs [20], so it is expected that this SNP should be replicated in the present study. For SNP rs1219648 at 10q26/*FGFR2*, association was observed in our previous study [25], the Carolina Breast Cancer Study (CBCS) [32], and the Women’s Health Initiative (WHI) study [35], but not in the Women’s Insights and Shared Experiences study [36]. SNP rs13387042 was replicated in our previous study [25] and in a consortium study of 3,016 cases and 2,745 controls [33], but not in the WHI study [35], the CBCS [32], nor another pooled study [34]. SNP rs8170 at 19p13/*BABAM1* was only investigated in a pooled study from Africans and AAs and no association was observed [34]. Both *CASP8* SNP (rs1045485) and *RAD51L* SNP (rs999737) have a MAF <5% in African-ancestry populations. Therefore, it is not surprising that these two SNPs were not replicated in all previous studies of African-ancestry populations [32]–[34]. The other four SNPs that showed associations with overall breast cancer in the present study, rs4849887, rs17817449, rs13329835 and rs4808801, were recently discovered [22] and have not been evaluated in previous studies of African-ancestry populations.

We did not replicate associations for the other 60 reported SNPs with the risk of overall breast cancer. Inconsistent results were reported for some of them in previous studies of African-ancestry populations. For example, SNP rs3803662 at 16q12/*TOX3* was replicated in the WHI [35], but not in the Black Women’s Health Study (BWHS) [37], the CBCS [32] and others [25], [34]. In addition, a significant association has been identified for this SNP with association in the opposite direction as previously reported [9]. SNP rs2981582 (10q26/*FGFR2*) was significantly associated with breast cancer risk in two studies [32], [33], but not in the other studies [25], [35], [36], [38]. The WHI study reported a significant association for rs10941679 at 5p12/*MRPS30* [35], and one study showed association for rs865686 at 9q31/*KLF4* [33], however, other studies did not replicate these two SNPs [25],[34],[39].

In general, our results that approximately 90% of index SNPs were not replicated in AAs are consistent with results from previous studies in African-ancestry populations [25], [32]–[39]. Rebbeck et al [36] did not find any association for three investigated SNPs. Huo et al [34] evaluated 19 genetic loci and none of them were replicated. Five of the seven investigated SNPs in the CBCS [32], 21 of the 22 investigated SNPs in the WHI [35], and 14 of 19 SNPs in a consortium study [33] were not replicated.

Because of the large difference in genetic architecture between African-ancestry and European/Asian ancestry populations, failure to replicate most of the reported SNPS in AAs is not surprising. Most, if not all, index SNPs identified in GWAS are associated with breast cancer risk through their strong LD with causal variants. African-ancestry populations have shorter LD and more genetic variations than European/Asian ancestry populations and may have different SNPs in LD with the causal variant. This may be the major reason why index SNPs are not replicated in African descendants. For example, in the BWHS, originally reported index SNPs rs10941679 and rs3803662 were not replicated, but other SNPs in these regions, rs16901937 and rs3104746, were associated with breast cancer [37], [39]. It has been reported that other markers were identified in AAs to better capture the association signal than the index SNPs originally discovered in the 2q35/*TNP1*, 5q11/*MAP3K1*, 10q26/*FGFR2,* and 19p13/*BABAM1* loci [33]. Second, allele frequencies for the index SNPs differ considerably across ethnic groups. Many index SNPs have lower MAF in AAs than in Europeans/Asians. Even if the effect size of the index SNP is the same across populations, larger sample size is required to detect association in AAs due to the lower MAF. Third, the vast majority of SNPs were originally discovered among European descendants, who have a much higher proportion of ER+ than ER- breast cancer. Because of this, most of the reported risk variants are, in general, more strongly associated with ER+ than ER- cancer [22]. African-ancestry women have a higher proportion of ER- breast cancer than European-ancestry women; this may be another reason for the non-replication in AAs.

To our knowledge, this is the first study in AAs that has evaluated index SNPs in all breast cancer susceptibility loci identified to date. However, the sample size in our study is relatively small, especially when stratified by breast cancer subtype. Some of the null associations observed in this study could be due to inadequate statistical power. Meta-analysis by pooling together all existing data in the AA populations will increase the statistical power to evaluate the effects of these variants in AAs. The other limitation of this study is that we only investigated index SNP in each locus. Large-scale fine mapping studies are needed to identify genetic risk variants at these loci in African-ancestry populations. Such work will be very helpful to identify causal variants for breast cancer.

In summary, in this African-ancestry population study, we replicated approximately 10% of index SNPs in 67 breast-cancer susceptibility loci. Heterogeneity was observed across the breast cancer subtype. These results show the complexity in applying GWAS findings to African-ancestry populations. Large-scale studies in AAs are needed to discover genetic risk variants which impact this population.

## Supporting Information

Table S1 **African genetic ancestry proportion of study participants estimated by AIMs.**
(DOCX)

Table S2 **Association of breast cancer risk with 60 SNPs located in reported breast-cancer susceptibility loci in African Americans with P>0.1.**
(DOCX)
