# Correction: Evaluating Genome-Wide Association Study-Identified Breast Cancer Risk Variants in African-American Women

**DOI:** 10.1371/annotation/54f35f2b-077c-4125-90f1-e550086938d5

**Published:** 2014-01-03

**Authors:** Jirong Long, Ben Zhang, Lisa B. Signorello, Qiuyin Cai, Sandra Deming-Halverson, Martha J. Shrubsole, Maureen Sanderson, Joe Dennis, Kyriaki Michailidou, Douglas F. Easton, Xiao-Ou Shu, William J. Blot, Wei Zheng

The name of the ninth author is spelled incorrectly. The correct name is: Kyriaki Michailidou.

