# Supplementary material for: Evaluating Genome-Wide Association Study-Identified Breast Cancer Risk Variants in African-American Women
Source: PLoS One. 2013 Apr 8;8(4):e58350. doi: 10.1371/journal.pone.0058350 (PMC3620157; doi:10.1371/journal.pone.0058350)
Supplement: Table S1 — African genetic ancestry proportion of study participants estimated by AIMs. (DOCX) [file pone.0058350.s001.docx]

| **Supplementary Table S1. African genetic ancestry proportion of study participants estimated by AIMs** | | | | | | |
| --- | --- | --- | --- | --- | --- | --- |
| Sample sources | Controls | All cases | ER+ | ER- | ER unknown | ER-/PR-/HER2- |
| NBHS | 80.14±12.05 | 81.22±10.82 | 79.65±11.73 | 80.58±10.32 | 82.48±10.23 | 80.72±10.99 |
| SCCS | 85.23±9.15 | 84.50±10.04 | 84.60±10.42 | 84.11±10.87 | 84.58±9.57 | 84.83±10.18 |
| Combined | 83.86±10.26 | 83.22±10.47 | 82.50±11.25 | 82.74±10.77 | 83.80±9.87 | 82.25±10.80 |
