# Supplementary material for: Evaluating Genome-Wide Association Study-Identified Breast Cancer Risk Variants in African-American Women
Source: PLoS One. 2013 Apr 8;8(4):e58350. doi: 10.1371/journal.pone.0058350 (PMC3620157; doi:10.1371/journal.pone.0058350)
Supplement: Table S2 — Association of breast cancer risk with 60 SNPs located in reported breast-cancer susceptibility loci in African Americans with P>0.1. (DOCX) [file pone.0058350.s002.docx]

| **Supplementary Table S2. Association of breast cancer risk with 60 SNPs located in reported breast-cancer susceptibility loci in African Americans with P>0.1** | | | | | | | |
| --- | --- | --- | --- | --- | --- | --- | --- |
| SNP | Chr./gene^a^ | Allele^b^ | RAF (cases /controls)^c^ | N (cases /controls) | OR (95% CI)^d^ | | *P*_trend_ |
|  |  |  |  |  | Heterozygous | Homozygous |  |
| rs11249433 | 1p11/FCGR1B | G/A | 0.107/0.108 | 1,112/930 | 0.92 (0.73-1.17) | 1.01 (0.45-2.27) | 0.574 |
| rs4973768 | 3p24/SLC4A7 | T/C | 0.357/0.385 | 1,113/929 | 0.88 (0.72-1.06) | 0.72 (0.54-0.95) | 0.019 |
| rs10941679 | 5p12/MRPS30 | G/A | 0.183/0.187 | 1,229/2,056 | 0.94 (0.79-1.10) | 1.10 (0.74-1.64) | 0.757 |
| rs889312 | 5q11/MAP3K1 | C/A | 0.346/0.333 | 1,229/2,058 | 0.86 (0.74-1.01) | 1.18 (0.93-1.51) | 0.857 |
| rs9485370 | 6q25/TAB2 | G/T | 0.794/0.788 | 1,109/929 | 1.13 (0.74-1.74) | 1.16 (0.77-1.76) | 0.533 |
| rs2046210 | 6q25/ESR1 | A/G | 0.623/0.621 | 1,230/2,057 | 0.94 (0.75-1.17) | 1.02 (0.81-1.28) | 0.643 |
| rs13281615 | 8q24/MYC | G/A | 0.453/0.460 | 1,216/2,049 | 1.07 (0.90-1.28) | 0.91 (0.74-1.12) | 0.486 |
| rs1011970 | 9p21/CDKN2A/2B | T/G | 0.327/0.313 | 1,113/930 | 1.12 (0.93-1.34) | 1.10 (0.81-1.50) | 0.305 |
| rs865686 | 9q31/KLF4 | T/G | 0.517/0.522 | 1,113/930 | 0.91 (0.73-1.13) | 0.97 (0.76-1.25) | 0.883 |
| rs2380205 | 10p15/ANKRD16 | C/T | 0.385/0.401 | 1,113/930 | 1.04 (0.86-1.27) | 0.77 (0.59-1.00) | 0.149 |
| rs10822013 | 10q21/ZNF365 | T/C | 0.231/0.227 | 1,113/930 | 1.00 (0.83-1.21) | 1.07 (0.71-1.62) | 0.834 |
| rs10995190 | 10q21/ZNF365 | G/A | 0.821/0.811 | 1,113/930 | 0.59 (0.34-1.01) | 0.71 (0.42-1.20) | 0.448 |
| rs704010 | 10q22/ZMIZ1 | T/C | 0.093/0.077 | 1,113/930 | 1.09 (0.84-1.40) | 2.90 (0.95-8.83) | 0.160 |
| rs2981582 | 10q26/FGFR2 | A/G | 0.489/0.473 | 1,230/2,057 | 1.15 (0.96-1.37) | 1.09 (0.88-1.35) | 0.395 |
| rs3817198 | 11p15/LSP1 | C/T | 0.160/0.161 | 1,209/2,054 | 0.99 (0.83-1.17) | 0.85 (0.52-1.38) | 0.646 |
| rs614367 | 11q13/CCND1 | T/C | 0.132/0.120 | 1,113/930 | 1.11 (0.89-1.38) | 1.16 (0.59-2.27) | 0.316 |
| rs10771399 | 12p11/PTHLH | A/G | 0.967/0.961 | 1,113/930 | NA | NA | 0.265 |
| rs1292011 | 12q24/MED13L | A/G | 0.552/0.566 | 1,113/930 | 0.99 (0.78-1.26) | 0.90 (0.70-1.16) | 0.370 |
| rs17271951 | 16q12/TOX3 | C/T | 0.064/0.058 | 1,113/930 | 1.00 (0.75-1.32) | 3.94 (0.46-33.42) | 0.699 |
| rs3803662 | 16q12/TOX3 | A/G | 0.517/0.523 | 1,228/2,059 | 1.02 (0.84-1.23) | 1.02 (0.83-1.26) | 0.857 |
| rs6504950 | 17q22/STXBP4 | G/A | 0.640/0.638 | 1,113/930 | 0.97 (0.74-1.27) | 1.01 (0.76-1.33) | 0.834 |
| rs2823093 | 21q21/NRIP1 | G/A | 0.555/0.565 | 1,113/930 | 0.90 (0.71-1.15) | 0.91 (0.70-1.17) | 0.507 |
| rs616488 | 1p36/PEX14 | A/G | 0.885/0.874 | 1,113/930 | 0.40 (0.15-1.07) | 0.51 (0.19-1.33) | 0.182 |
| rs11552449 | 1p13/AP4B1 | T/C | 0.034/0.037 | 1,113/930 | 0.90 (0.63-1.29) | 0.65 (0.04-11.30) | 0.544 |
| rs2016394 | 2q31/DLX2 | G/A | 0.738/0.731 | 1,091/919 | 1.26 (0.86-1.83) | 1.27 (0.88-1.84) | 0.367 |
| rs1550623 | 2q31/CDCA7 | A/G | 0.704/0.703 | 1,113/929 | 0.98 (0.71-1.36) | 0.98 (0.71-1.35) | 0.907 |
| rs16857609 | 2q35/DIRC3 | T/C | 0.257/0.234 | 1,113/930 | 1.12 (0.93-1.36) | 1.25 (0.86-1.83) | 0.120 |
| rs6762644 | 3p26/ITPR1 | G/A | 0.476/0.466 | 1,113/930 | 1.21 (0.98-1.49) | 1.12 (0.87-1.43) | 0.312 |
| rs12493607 | 3p24/TGFBR2 | C/G | 0.137/0.125 | 1,113/930 | 1.11 (0.89-1.37) | 1.15 (0.58-2.27) | 0.330 |
| rs9790517 | 4q24/TET2 | T/C | 0.068/0.057 | 1,113/929 | 1.10 (0.83-1.45) | 3.71 (0.42-32.35) | 0.323 |
| rs6828523 | 4q34/ADAM29 | C/A | 0.622/0.615 | 1,113/930 | 1.05 (0.80-1.37) | 1.05 (0.80-1.39) | 0.741 |
| rs10472076 | 5q11/RAB3C | C/T | 0.258/0.269 | 1,112/930 | 0.95 (0.79-1.14) | 0.87 (0.60-1.25) | 0.388 |
| rs1353747 | 5q11/PDE4D | T/G | 0.985/0.988 | 1,113/930 | 1.29 (0.74-2.25) | NA | 0.373 |
| rs1432679 | 5q33/EBF1 | C/T | 0.817/0.822 | 1,111/930 | 1.22 (0.72-2.08) | 1.20 (0.71-2.03) | 0.803 |
| rs11242675 | 6p25/FOXQ1 | T/C | 0.502/0.499 | 1,072/913 | 0.91 (0.73-1.13) | 1.01 (0.79-1.31) | 0.923 |
| rs204247 | 6p23/RANBP9 | G/A | 0.343/0.346 | 1,113/930 | 1.02 (0.85-1.23) | 0.94 (0.71-1.26) | 0.840 |
| rs17529111 | 6q14/FAM46A | C/T | 0.064/0.059 | 1,113/930 | 1.02 (0.77-1.34) | 1.22 (0.22-6.89) | 0.862 |
| rs720475 | 7q35/ARHGEF5 | G/A | 0.893/0.888 | 1,113/930 | 1.16 (0.47-2.87) | 1.22 (0.50-2.97) | 0.565 |
| rs9693444 | 8p21/RPL17P33 | A/C | 0.392/0.381 | 1,113/930 | 1.02 (0.84-1.23) | 1.09 (0.83-1.43) | 0.565 |
| rs6472903 | 8q21/HNF4G | T/G | 0.905/0.900 | 1,110/928 | 0.78 (0.31-1.95) | 0.85 (0.35-2.07) | 0.666 |
| rs2943559 | 8q21/HNF4G | G/A | 0.230/0.221 | 1,098/918 | 0.94 (0.78-1.14) | 1.45 (0.95-2.20) | 0.506 |
| rs11780156 | 8q24/MYC | T/C | 0.039/0.038 | 1,112/930 | 1.05 (0.74-1.50) | 0.15 (0.02-1.46) | 0.753 |
| rs10759243 | 9q31/KLF4 | A/C | 0.609/0.633 | 1,113/930 | 1.10 (0.84-1.43) | 0.92 (0.70-1.21) | 0.272 |
| rs7072776 | 10p12/MLLT10 | A/G | 0.516/0.516 | 1,112/930 | 1.08 (0.87-1.35) | 1.01 (0.78-1.30) | 0.976 |
| rs11814448 | 10p12/DNAJC1 | C/A | 0.650/0.658 | 1,113/930 | 1.11 (0.83-1.48) | 1.02 (0.76-1.37) | 0.800 |
| rs7904519 | 10q25/TCF7L2 | G/A | 0.809/0.802 | 1,110/929 | 0.87 (0.54-1.41) | 0.95 (0.59-1.51) | 0.630 |
| rs11199914 | 10q26/FGFR2 | C/T | 0.462/0.471 | 1,113/930 | 1.03 (0.83-1.26) | 0.92 (0.71-1.18) | 0.548 |
| rs3903072 | 11q13/OVOL1 | G/T | 0.841/0.844 | 1,113/930 | 1.10 (0.63-1.92) | 1.08 (0.63-1.86) | 0.969 |
| rs11820646 | 11q24/BARX2 | C/T | 0.765/0.787 | 1,113/930 | 1.09 (0.72-1.64) | 0.92 (0.62-1.36) | 0.150 |
| rs12422552 | 12p13/ATF7IP | C/G | 0.428/0.419 | 1,098/917 | 0.98 (0.81-1.20) | 1.08 (0.83-1.41) | 0.653 |
| rs17356907 | 12q22/NTN4 | A/G | 0.793/0.790 | 1,113/925 | 1.60 (1.04-2.48) | 1.44 (0.95-2.20) | 0.753 |
| rs11571833 | 13q13/BRCA2 | T/A | 0.001/0.002 | 1,113/930 | 0.71 (0.14-3.59) | NA | 0.678 |
| rs2236007 | 14q13/PAX9 | G/A | 0.928/0.930 | 1,109/930 | 0.82 (0.23-2.92) | 0.86 (0.25-3.01) | 0.807 |
| rs2588809 | 14q24/RAD51L1 | T/C | 0.289/0.298 | 1,113/930 | 0.96 (0.79-1.15) | 0.97 (0.70-1.35) | 0.689 |
| rs941764 | 14q32/CCDC88C | G/A | 0.736/0.727 | 1,113/928 | 0.95 (0.67-1.35) | 1.07 (0.76-1.50) | 0.334 |
| rs527616 | 18q11/AQP4 | G/C | 0.866/0.879 | 1,113/930 | 0.91 (0.47-1.77) | 0.83 (0.44-1.60) | 0.351 |
| rs1436904 | 18q11/CHST9 | T/G | 0.745/0.753 | 1,113/930 | 0.74 (0.50-1.10) | 0.78 (0.54-1.15) | 0.675 |
| rs3760982 | 19q13/KCNN4 | A/G | 0.470/0.469 | 1,110/929 | 1.10 (0.90-1.36) | 1.01 (0.79-1.31) | 0.841 |
| rs132390 | 22q12/EMID1 | C/T | 0.060/0.060 | 1,113/930 | 1.00 (0.76-1.32) | 1.46 (0.24-8.80) | 0.919 |
| rs6001930 | 22q13/MKL1 | C/T | 0.129/0.151 | 1,112/930 | 0.77 (0.63-0.95) | 0.90 (0.50-1.62) | 0.035 |
| ^a^ The closest gene. | | | | | | | |
| ^b^ Risk/reference alleles based on NCBI Human Genome Build 36 forward strand. | | | | | | | |
| ^c^ Risk allele frequency of cases and controls. | | | | | | | |
| ^d^ Adjusted with age, study (NBHS and SCCS), and the first ten principal components. | | | | | | | |
